# Comparing the Bacterial Community in the Gastrointestinal Tracts Between Growth-Retarded and Normal Yaks on the Qinghai–Tibetan Plateau

**DOI:** 10.3389/fmicb.2020.600516

**Published:** 2020-12-18

**Authors:** Jian Ma, Yixiao Zhu, Zhisheng Wang, Xiong Yu, Rui Hu, Xueying Wang, Guang Cao, Huawei Zou, Ali Mujtaba Shah, Quanhui Peng, Bai Xue, Lizhi Wang, Suonan Zhao, Xiangying Kong

**Affiliations:** ^1^Low Carbon Breeding Cattle and Safety Production University Key Laboratory of Sichuan Province, Animal Nutrition Institute, Sichuan Agricultural University, Chengdu, China; ^2^College of Animal Science, Xinjiang Agricultural University, Urumchi, China; ^3^Haibei Demonstration Zone of Plateau Modern Ecological Animal Husbandry Science and Technology, Haibei, China

**Keywords:** yak, growth retardation, bacterial community, gastrointestinal tract, Qinghai–Tibetan Plateau

## Abstract

In ruminants, the bacterial community in the gastrointestinal tract (GIT) has an essential role in healthy growth. Examining the bacterial composition in the GIT between growth-retarded and normal yaks could improve our understanding of the role of microorganisms in yaks with growth retardation. In this study, eight male yaks with growth retardation were used as the growth-retarded yak (GRY) group, and another eight male growth normal yaks (GNYs) with the same breed and age were used as the GNY group. We compared the bacterial community in the rumen, duodenum, jejunum, ileum, cecum, and colon between GRY and GNY groups based on the 16S ribosomal RNA gene sequencing. Alpha-diversity revealed that the Shannon index in the duodenum and ileum of the GNY group was higher (*P* < 0.05) than that of the GRY group. However, the opposite trend was found in the jejunum and cecum. The principal coordinates analysis (PCoA) showed that the bacterial structure in all segments of GIT differed from each other between two groups. In the rumen, the relative abundances of *Ruminococcaceae NK4A214* group, *Ruminococcaceae UCG-014*, and *Treponema 2* were higher (*P* < 0.05) in the GNY group as compared with the GRY group. However, the *Christensenellaceae R-7* group exhibited an opposite trend. In the jejunum, compared with the GNY group, the *unclassified Chitinophagaceae* was enriched significantly (*P* < 0.05) in the GRY group. However, the *unclassified Peptostreptococcaceae*, *Christensenellaceae R-7* group, and *Lachnospiraceae NK3A20* group were enriched (*P* < 0.05) in the GNY group. In the ileum, the relative abundances of *the Rikenellaceae RC9 gut* group and *Prevotellaceae UCG-004* were higher (*P* < 0.05) in the GNY group than those in the GRY group. In the cecum, the GNY group showed a higher (*P* < 0.05) relative abundance of *Prevotellaceae UCG-003* as compared with the GRY group. In the colon, the relative abundances of *Treponema 2* and *unclassified Lachnospiraceae* were slightly higher (0.05 < *P* < 0.10) in the GNY group than those in the GRY group. Overall, these results improve our knowledge about the bacterial composition in the GIT of growth-retarded and normal yaks, and regulating the bacterial community may be an effective solution to promote the compensatory growth of GRYs.

## Introduction

The yak (*Bos grunniens*) is a major indigenous ruminant inhabiting extreme climate on the Qinghai–Tibetan Plateau at high altitudes from 2,500 to 6,000 m. Most of the yaks in the world are distributed in China, and the milk, meat, fur, and fuel (feces for living fuel) of yaks are used by local Tibetan herdsmen for the major living resources and financial income (Xu T. et al., 2017). In addition, yaks remain semidomesticated status, grazing on the grassland with a natural mating, and have a crucial ecological niche in the Qinghai–Tibetan Plateau ecosystem (Zhang et al., 2016). However, in the Qinghai–Tibetan Plateau, the climatic environment is sharp frost (average temperature −15 to −5°C) with heavy snowfall in the long-term cold season (from October to May). In the cold season, due to the grass is snow-covered and withered, the grassland of the plateau is extremely short of forage. On the other hand, the seasonal reproduction characteristics of yaks, which usually mate from June to October and have a delivery during May to September after a 265 day pregnancy (Zi, 2003), lead to severe malnutrition of yak calves in early life. Previous studies have reported that severe malnutrition of animals during early age restrained normal growth and development in the future (Du et al., 2010; Gu et al., 2017). Therefore, the yaks with growth retardation widely exist on the Qinghai–Tibetan Plateau.

Our previous studies found that compared with growth normal yaks (GNYs), growth-retarded yaks (GRYs) had lower body weight (BW) and feed efficiency and higher morbidity and mortality, so that the economic income of yaks farming was reduced. Additionally, the papillae height of rumen and the ruminal and intestinal weight in GNYs were lower than those in GRYs, indicating that the development of gastrointestinal tract (GIT) of GRYs was dysplastic (Hu et al., 2016, 2019). As important organs for feed digestion, nutrients absorption, endocrine, and immune functions, healthy gastrointestinal development is the foundation of animals’ body nutrients deposition and growth (Celi et al., 2017; Powell et al., 2017). In ruminants, the rumen is a special digestive organ for digesting roughage, and its healthy development has a critical role in the process of digestion, particularly for yaks (digestion of natural grass) (Zhou et al., 2011). Moreover, studies have shown that severe malnutrition of animals during a young age could damage the structure and function of the GIT and then result in subsequent growth retardation (Li et al., 2018; Zhang et al., 2018).

The microbial community in the GIT of mammalian animals has been increasingly identified as an important factor in animals’ growth, health, and production performance (Holmes et al., 2012; Yeoman and White, 2014). The microflora in the GIT is an intricate micro-ecosystem, which mainly composes of ciliate protozoa, anaerobic fungi, archaea, and bacteria (Falony et al., 2016; Cui et al., 2020a). A previous study has found that the microbiota acted as an essential role in regulating homeostasis and nutritional metabolism of the host’s GIT (Rey et al., 2013). Furthermore, symbiotic relationships between the host and microflora in the GIT have been demonstrated to increase the host’s resistibility to external pathogenic bacteria and then to crucially facilitate the maturation of the immune system (Dolan and Chang, 2016; Parker et al., 2017). As metabolites of microorganisms, the butyrate and propionate are deemed important modulatory media (Ohland and Jobin, 2015). In ruminants, microbial fermentation is an important process to digest high fiber feedstuff. The typical example in the rumen is the fibro lytic activity, which contributes to the conversion of crude fiber to volatile fatty acids (VFAs), mainly including acetate, propionate, and butyrate (Jami et al., 2013). As the primary energy substrate, VFAs are produced in the rumen by microbial fermentation and then quickly absorbed by the ruminal epithelium and provide approximately 80% of metabolizable energy for the ruminants (Gozho and Mutsvangwa, 2008). Our previous study found that the GRYs had lower concentrations of butyrate and propionate in the rumen as compared with normal yaks (Hu et al., 2019). Another study in beef calves reported that the relative abundances of *Proteobacteria*, *Rhodospirillaceae*, *Campylobacterales*, and *Butyricimonas*, which had a vital function in the production of energy and VFA, were decreased in the fecal microbiota of growth-retarded calves compared with normal calves. On the contrary, the relative abundances of suspected pathogens, including *Anaeroplasma* and *Acholeplasma*, were increased in growth-retarded calves (Du et al., 2018).

According to the studies mentioned earlier, we can find that the gastrointestinal microflora plays an important role in the healthy growth of animals. On the other hand, because of the convenience of fecal sampling and conventional viewpoint in the rumen as the main functional organ of ruminants, the majority of researchers paid more attention to the bacterial community in the rumen and feces (Wang et al., 2016; Xu H. et al., 2017). However, numerous researches reported that the ruminal or fecal microbial community could not represent all the GIT (de Oliveira et al., 2013; Zhao et al., 2015). Therefore, in the current study, we compared the bacterial community in the rumen, duodenum, jejunum, ileum, cecum, and colon of GRYs and GNYs based on the bacterial 16S ribosomal RNA (rRNA) gene sequencing to improve our understanding of the role of microflora in the nutrition and metabolism of yaks and provide new insights into scientific management by regulating the microbial community of growth-retarded animals.

## Materials and Methods

### Ethics Statement

This animal experiment was carried out in accordance with the Regulation on the Administration of Laboratory Animals (2017 Revision) promulgated by Decree No. 676 of the State Council. The protocol was authorized and approved by the Institutional Animal Care and Use Committee of Sichuan Agricultural University (Chengdu, Sichuan, China).

### Animals and Experimental Design

The study was conducted at the farm of Animal Husbandry and Veterinary Institute of Haibei Prefecture, Qinghai Province, China (altitude approximately 3,200 m; 100°54′ E longitude and 36°57′ N latitude). According to the previous studies on BW of yaks population by our group (Hu et al., 2016, 2019) and investigation results of local yaks population by the Animal Husbandry and Veterinary Institute of Haibei Prefecture, growth retardation is defined as BW that the BW of one yak with the same breed and age is in the low 10% of yaks population in the absence of deformity or early disease signs (Jones et al., 2012). In this experiment, eight male Qinghai Plateau yaks (BW = 74.00 ± 6.41 kg and age = 16 months old) with growth retardation were used as the GRY group. Another eight male GNYs (BW = 111.63 ± 4.03 kg) with the same breed and age were selected as the GNY group. The detailed BW of yaks is presented in Supplementary Table 1. All the yaks were pastured on the same plateau grassland without supplementary feed and housing. After the yaks were marked with ear tags, a 15 day observation period followed by the sample collection was implemented.

### Sample Collection

Twelve yaks from two groups, which were close to the group average BW, were slaughtered by captive bolt stunning and exsanguinated humanely. The process of slaughter followed the National Standard Operating Procedures (GB/T 19477-2004, cattle slaughtering, China). After slaughter, the abdominal cavity was opened, and then, the different gastrointestinal segments, including rumen, duodenum, jejunum, ileum, cecum, and colon, were isolated with a suture line to avoid reflux of digesta among adjacent regions. The digesta samples in different GIT were collected and put into the sterile centrifuge tubes, individually. Finally, the digesta samples were immediately frozen in liquid nitrogen and then stored at −80°C until the total DNA extraction.

### Microbial DNA Isolation

All the digesta samples were thawed (4°C), and the digesta samples (200 mg) in each GIT were used for total genomic DNA isolation using the TIANamp Stool DNA Kit (TIANGEN, Beijing, China). The concrete operating steps of microbial DNA extraction were according to the manufacturer’s instructions with the bacterial lysis step, bead-beating step using a TGrinder H24 Tissue Homogenizer (TIANGEN, Beijing, China). Subsequently, the DNA purification was processed with a spin column. The concentration and purity of DNA were assessed using 0.8% Agarose Gel Electrophoresis and a NanoDrop 2,000 Spectrophotometer (Thermo Scientific, Waltham, MA, United States). Finally, DNA was diluted to 10 ng/μl using sterile ultrapure water and stored at −80°C for a downstream procedure.

### PCR Amplification

The universal primers 515F (5′-GTGCCAGCMGCCGCGGTAA-3′) and 806R (5′-GGACTACHVGGGTWTCTAAT-3′) with 12 nt unique barcodes were used to amplify the V4 variable region of the 16S rRNA gene from all DNA samples (Caporaso et al., 2011). The PCR reactions were carried out in a 25 μl mixture containing 1 × PCR buffer, 1.5 mmol/L MgCl_2_, 0.4 μmol/L deoxynucleoside triphosphates, each primer at 1.0 μmol/L, 0.5 U of KOD-Plus-Neo DNA Polymerase (TOYOBO, Osaka, Japan), and 10-ng template DNA. The PCR amplification was performed using Applied Biosystems^®^ Gene Amp^®^ PCR System 9,700 (Thermo Scientific, Waltham, MA, United States) according to the following process: initial denaturation at 94°C for 1 min, followed by 30 cycles (denaturation at 94°C for 20 s, annealing at 54°C for 30 s, and extension at 75°C for 30 s), and a final extension at 72°C for 5 min. Three PCR replicates were performed for each sample, and the three replicates per sample for PCR reactions were combined together.

PCR products mixed with 1/6 volume of 6 × loading buffer were loaded on 2% agarose gel for detection. Samples with a bright main strip of approximately 410 bp were chosen for further analysis of purification and quantification. The electrophoresis band was purified via QIAquick Gel Extraction Kit (QIAGEN, Adelaide, SA, Australia). DNA was quantified using Qubit@ 2.0 Fluorometer (Thermo Scientific, Waltham, MA, United States) reference to the electrophoresis preliminary test results. Finally, PCR products from all samples were pooled with an equal molar amount for subsequent sequencing analysis.

### High-Throughput Sequencing and Sequencing Data Analysis

Sequencing libraries were generated using TruSeq DNA PCR-Free Sample Prep Kit (Illumina, San Diego, CA, United States) according to the manufacturer’s recommendations, and index codes were added. The library quality was assessed by the Qubit@ 2.0 Fluorometer (Thermo Fisher Scientific, Waltham, MA, United States) and Agilent Bioanalyzer 2,100 system (Agilent Technologies, Carpinteria, CA, United States). Then, the library was sequenced on an Illumina HiSeq 2500 platform (Illumina, San Diego, CA, United States) by 2 × 250 bp paired-end sequencing.

Paired-end reads from the original DNA fragments were merged using FLASH (version 1.03) (Magoč and Salzberg, 2011). Each sample sequence was split from the reads based on the unique Barcodes, and then, the raw data were obtained by truncating the Barcodes sequence. The quality control was conducted via Trimmomatic (version 0.36) (Bolger et al., 2014). Then, the Uchime algorithm was used to remove the chimeras reference to the Gold database to get the clean reads (Edgar et al., 2011). Subsequently, sequences were clustered into operational taxonomic units (OTUs) at 97% identity threshold using UPARSE algorithms in Usearch software (version 8.0), and the representative sequence with the most abundant sequence was selected for each OTU (Edgar, 2013). The assignments of taxonomic OTU were performed using the UCLUST (Edgar, 2010) and Silva database (version 132) (Quast et al., 2013). The PyNAST was used to compare and filter the representative sequences (Gregory et al., 2010). All samples were homogenized, and the samples with the least amount of data were taken as the standard for resampling to avoid the effects of sequencing depth on community diversity.

According to the operations mentioned earlier, the sequencing data analysis was carried out by R software (version 3.5.3). The Vegan was used to calculate the alpha- and beta-diversity parameters. In addition, rarefaction curves were generated based on the number of OTUs. The distance of Bray–Curtis was calculated via the vegdist function of Vegan. The PCoA based on Bray–Curtis dissimilarity matrices was analyzed using the ape package, and the adonis function of Vegan was used to calculate the permutational multivariate analysis of variance. The heat map was obtained with the dominant bacteria using the z-score normalization for each sample [z score = (actual relative abundance of a genus − mean relative abundance of the same genus)/standard deviation]. The heat map and bubble plot were generated using the online resources ImageGP^1^. Besides, the linear discriminant analysis effect size (LEfSe) method (Segata et al., 2011) was performed to identify bacterial taxa with significant differences between the GNY and GRY groups using the online resources^2^. In our study, the linear discriminant analysis score ≥ 3.5 was identified as an important contributor to each group (Mörkl et al., 2017).

### Statistical Analysis

All statistical analysis was performed using the SPSS software (version 20.0 for Windows, SPSS, Chicago, IL, United States). All data were firstly performed normality and homogeneity of variances tests. Alpha-diversity indexes and the bacterial relative abundance between GNY and GRY groups were analyzed using the *t*-test. If the data did not satisfy normal distribution or homogeneity of variance, these data were analyzed using the non-parametric test. Data were presented as mean and standard error of the mean. The significance level of alpha-diversity indexes was indicated at *P*-value < 0.05, and a trend was declared at 0.05 ≤ *P*-value < 0.10. *P*-values of < 0.05 after false discovery rate correction of bacterial composition using the Benjamini–Hochberg procedure for the multiple comparisons were considered significant, and a trend was declared at 0.05 ≤ *P*-value < 0.10.

## Results

### Data Acquisition and Analysis

**FIGURE 1:**
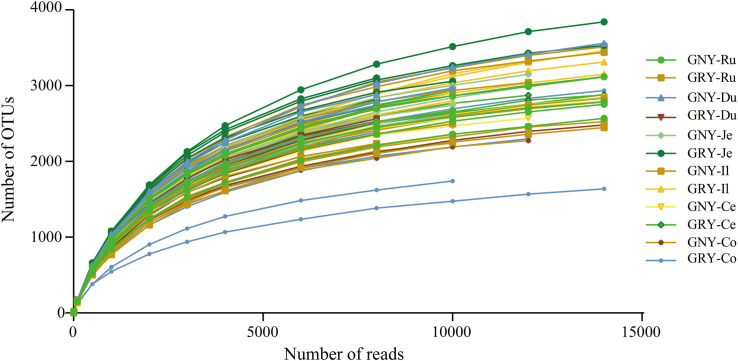
Rarefaction curves for all gastrointestinal digesta samples (*n* = 72). Operational taxonomic units were assigned at the 97% sequence similarity level. GNY, growth normal yak (*n* = 6); GRY, growth-retarded yak (*n* = 6); Ru, rumen; Du, duodenum; Je, jejunum; Il, ileum; Ce, cecum; Co, colon.

In the current study, 72 samples from the digesta of rumen, duodenum, jejunum, ileum, cecum, and colon were collected from two groups. We obtained a total of 2,476,770 raw sequences by 16S rRNA gene sequencing, with an average of 34,399 ± 2,926 (mean ± standard error) sequences per sample. After quality filtering of the sequence, the 2,361,454 valid sequences were obtained, with an average of 32,798 ± 2,801 sequences per sample (Supplementary Table 2). The average sequencing length of all samples was 298 ± 4.25 bp. Based on a 97% nucleotide sequence identity between reads, we identified 8,398, 10,436, 8,130, 6,882, 8,158, and 7,817 OTUs in the rumen, duodenum, jejunum, ileum, cecum, and colon, respectively. The Q30 (Supplementary Table 2) and rarefaction curves (Figure 1) were generated for each sample to evaluate whether sampling provided sufficient OTU coverage to accurately describe the bacterial community of each region. The curves of all samples reached a plateau, suggesting that a sufficient number of sequences had been generated to investigate bacterial diversity in the rumen, duodenum, jejunum, ileum, cecum, and colon.

### Difference of Bacterial Alpha-Diversity Indexes in the Gastrointestinal Tract Between Growth-Retarded and Normal Yaks

Alpha-diversity analysis showed that the observed species, Chao1, ACE, and Shannon indexes in the rumen and colon were similar (*P* > 0.05) between the GNY and GRY groups (Table 1). In the duodenum and ileum, the Shannon index of the GNY group was higher (*P* < 0.05) than that of the GRY group. However, the opposite trend was observed in the jejunum and cecum. No significant difference (*P* > 0.05) of observed species, Chao1, and ACE indexes in the duodenum and ileum was found between them. However, in the GNY group, the observed species, Chao1, and ACE indexes of jejunum and cecum were lower (*P* < 0.05) than the GRY group (Table 1).

**TABLE 1:** Comparison of the alpha-diversity indexes in the different gastrointestinal regions of yaks.

Regions	Indexes	GNY	GRY	SEM	*P*-value
Rumen	Observed species	2,197	2,071	70.6	0.397
	Chao1	2,955	2,731	112	0.339
	ACE	3,115	2,845	119	0.273
	Shannon	6.53	6.43	0.069	0.476
Duodenum	Observed species	2,674	2,392	88.0	0.112
	Chao1	3,707	3,306	158	0.220
	ACE	4,022	3,567	186	0.237
	Shannon	7.00	6.76	0.059	0.033
Jejunum	Observed species	2,402	2,932	103	0.003
	Chao1	3,103	3,859	191	0.040
	ACE	3,240	4,087	216	0.043
	Shannon	6.50	7.05	0.097	0.001
Ileum	Observed species	2,281	2,369	48.5	0.387
	Chao1	3,034	3,544	159	0.110
	ACE	3,157	3,789	183	0.083
	Shannon	6.82	6.36	0.080	< 0.001
Cecum	Observed species	1,980	2,419	73.0	< 0.001
	Chao1	2,616	3,304	118	< 0.001
	ACE	2,725	3,487	128	< 0.001
	Shannon	6.45	6.76	0.061	0.003
Colon	Observed species	2,054	1,750	118	0.212
	Chao1	2,843	2,429	186	0.286
	ACE	3,043	2,606	208	0.315
	Shannon	6.49	6.05	0.134	0.102

*GNY, growth normal yak (n = 6); GRY, growth-retarded yak (n = 6); SEM, standard error of the mean.*

### Difference of Bacterial Beta-Diversity in the Gastrointestinal Tract Between Growth-Retarded and Normal Yaks

**FIGURE 2:**
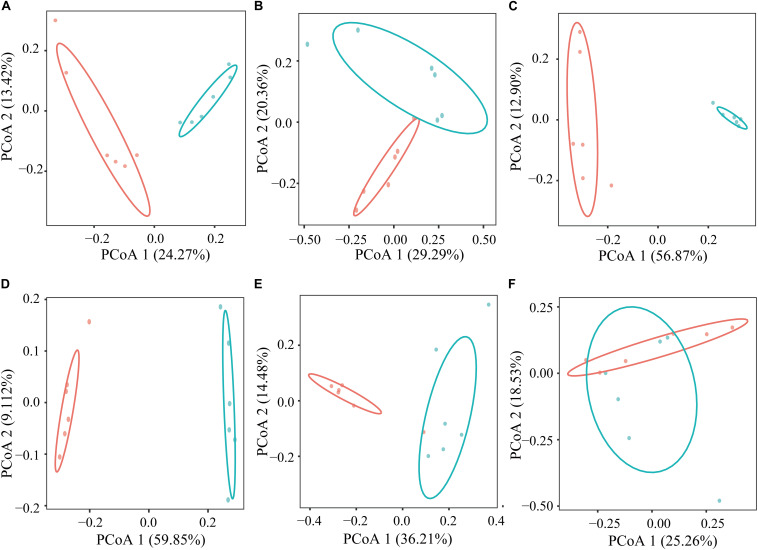
Principal coordinates analysis of bacterial communities in the rumen **(A)**, duodenum **(B)**, jejunum **(C)**, ileum **(D)**, cecum **(E)**, and colon **(F)** between GNY (*n* = 6) and GRY (*n* = 6) groups based on the Bray–Curtis distance. GNY, growth normal yak (red color); GRY, growth-retarded yak (blue color).

In the current study, based on the Bray–Curtis dissimilarity matrices, when using the PCoA to examine the structure of microflora in the GIT between the GNY and GRY groups, the bacterial communities in the rumen (Figure 2A), duodenum (Figure 2B), jejunum (Figure 2C), ileum (Figure 2D), and cecum (Figure 2E) were clearly separated from each other. Meanwhile, the permutational multivariate analysis of variance was used to determine whether there is a significant difference of distance in the GIT between the GNY and GRY groups. The results showed significant differences in the rumen (*R*^2^ = 0.218, *P* = 0.003), duodenum (*R*^2^ = 0.183, *P* = 0.010), jejunum (*R*^2^ = 0.557, *P* = 0.003), ileum (*R*^2^ = 0.594, *P* = 0.003), and cecum (*R*^2^ = 0.304, *P* = 0.003) between the two groups (Supplementary Table 3). Besides, in the colon (Figure 2F), the bacterial community had a trend of difference between the two groups (*R*^2^ = 0.144, *P* = 0.051) (Supplementary Table 3).

### Difference of Bacterial Community in the Rumen Between Growth-Retarded and Normal Yaks

**FIGURE 3:**
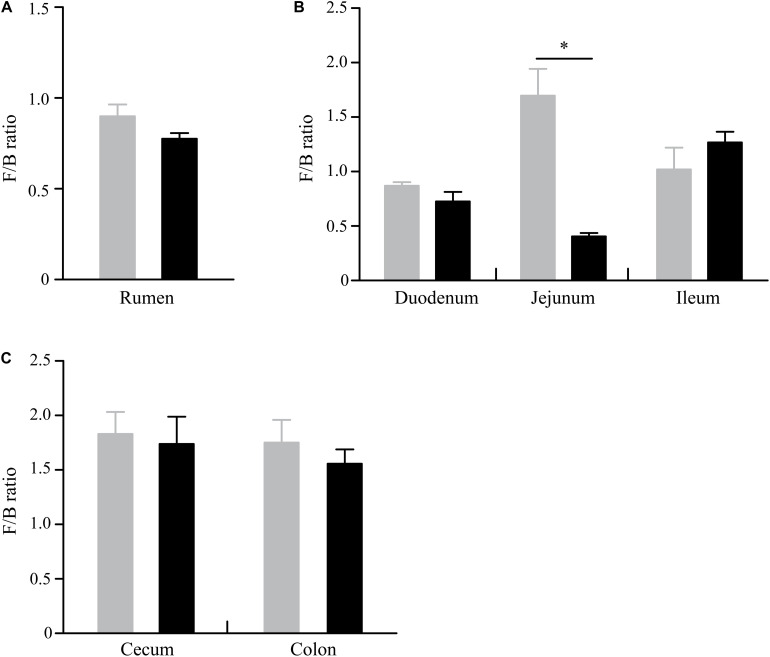
Difference of Firmicutes-to-Bacteroidetes ratio in the rumen **(A)**, small intestine **(B)**, and large intestine **(C)** between GNY (*n* = 6) and GRY (*n* = 6) groups. GNY, growth normal yak (gray bar); GRY, growth-retarded yak (black bar). The asterisk indicates a significant difference between GNY and GRY groups (*P* < 0.05).

At the phylum level, a total of 32 phyla were identified from 12 digesta samples in the rumen, and the number of bacteria phyla detected in the GNY and GRY groups were 29 and 31, respectively. The Bacteroidetes (GNY = 45.23% and GRY = 44.81%) and Firmicutes (GNY = 40.24% and GRY = 34.60%) were the predominant bacteria in the rumen of yaks, accounting for more than 75% of the total relative abundance of total bacteria. Compared with the GNY group, the relative abundance of Spirochetes of the GRY group was lower (*P* = 0.033). In contrast, the relative abundances of Actinobacteria and Patescibacteria were higher (*P* < 0.05) in the GRY group than those in the GNY group. Besides, the relative abundances of Firmicutes (*P* = 0.052) and Tenericutes (*P* = 0.072) tended to be higher in the GNY group than the GRY group. An opposite trend of Chloroflexi was found between the two groups (Table 2). No obvious difference (*P* > 0.05) of Firmicutes-to-Bacteroidetes ratio (F/B ratio) was found between the two groups (Figure 3A).

**TABLE 2:** Comparison of the relative abundance (%) of the representative bacteria at the phylum level in the rumen of growth-retarded and normal yaks.

Items	GNY	GRY	SEM	*P*-value
Bacteroidetes	45.23	44.81	0.954	0.840
Firmicutes	40.24	34.60	1.336	0.052
Spirochetes	3.41	1.74	0.356	0.033
Chloroflexi	1.08	2.15	0.249	0.060
Tenericutes	1.00	0.72	0.074	0.072
Proteobacteria	0.73	1.50	0.269	0.205
Actinobacteria	0.43	1.42	0.166	0.008
Patescibacteria	0.42	0.73	0.060	0.015
Planctomycetes	0.43	0.74	0.103	0.180
Kiritimatiellaeota	0.51	0.46	0.030	0.528

*GNY, growth normal yak (n = 6); GRY, growth-retarded yak (n = 6); SEM, standard error of the mean. The phylum with the average relative abundance was ≥ 0.5% in at least one group.*

**FIGURE 4:**
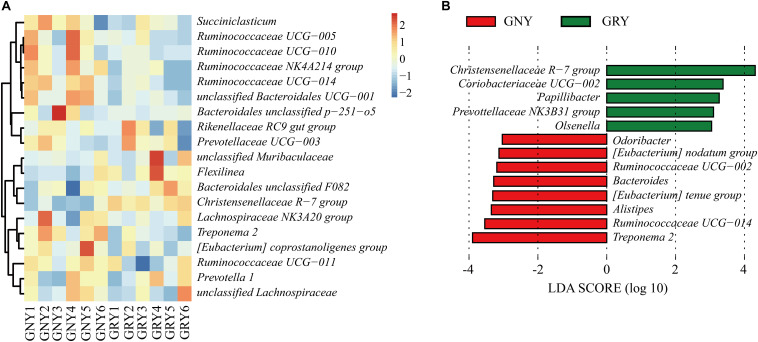
Taxa plots depicting the ruminal bacterial composition of GNY (*n* = 6) and GRY (*n* = 6) groups. **(A)** Heat map showing the relative abundance of dominant bacteria at genus level in the rumen. Genus with the average relative abundance was ≥ 1% in at least one group. **(B)** Linear discriminant analysis effect size analysis identified the most differentially abundant genera between GNY (red) and GRY (green) groups. Genus with linear discriminant analysis values higher than 3.5 is displayed. GNY, growth normal yak; GRY, growth-retarded yak.

At the genus level (Figure 4A and Supplementary Table 4), *Rikenellaceae RC9 gut* group was the most dominant bacterium in the ruminal digesta of the two groups (GNY = 13.94% and GRY = 13.95%), followed by the Bacteroidales unclassified F082 (GNY = 8.56% and GRY = 10.23%), *Christensenellaceae R-7* group (GNY = 6.17% and GRY = 9.40%), and *Prevotella 1* (GNY = 6.22% and GRY = 6.19%). The relative abundances of *Ruminococcaceae NK4A214* group, *Ruminococcaceae UCG-014*, and *Treponema 2* were higher (*P* < 0.05) in the GNY group than those in the GRY group. Compared with the GNY group, a higher (*P* < 0.05) relative abundance of *Christensenellaceae R-7* group was found in the GRY group. Furthermore, the relative abundance of *unclassified Bacteroidales UCG-001* in the GNY group was slightly higher (*P* = 0.084) than that in the GRY group. By using LEfSe analysis, the differential genera were further identified based on the relative abundance of different bacteria. In the rumen, 13 differential bacteria, mainly including *Christensenellaceae R-7 group*, *Treponema 2*, and *Ruminococcaceae UCG-014*, were identified between the two groups (Figure 4B).

### Difference of Bacterial Community in the Small Intestine Between Growth-Retarded and Normal Yaks

In the small intestine, we observed a total of 37 phyla in 36 digesta samples. The number of bacteria phyla identified in the duodenum, jejunum, and ileum of the GNY and GRY groups were 31 and 30, 30 and 31, and 30 and 32, respectively. The Firmicutes (duodenum: GNY = 36.58% and GRY = 34.04%; jejunum: GNY = 38.94% and GRY = 15.18%; ileum: GNY = 35.01% and GRY = 38.03%), Bacteroidetes (duodenum: GNY = 42.15% and GRY = 48.36%; jejunum: GNY = 24.89% and GRY = 37.67%; ileum: GNY = 34.39% and GRY = 27.86%), and Proteobacteria (duodenum: GNY = 5.77% and GRY = 4.40%; jejunum: GNY = 9.49% and GRY = 17.81%; ileum: GNY = 12.28% and GRY = 14.13%) were the dominant bacteria in each segment of small intestine. In the duodenum, the GNY group showed a slightly higher (*P* = 0.055) relative abundance of Planctomycetes as compared with the GRY group. In the jejunum, the relative abundance of Bacteroidetes in the GRY group was higher (*P* < 0.05) than that in the GNY group. However, an opposite tendency was found in the ileum. In the jejunum, the relative abundances of Proteobacteria, Acidobacteria, Planctomycetes, Actinobacteria, and Gemmatimonadetes in the GRY group were higher (*P* < 0.05) than those in the GNY group (Table 3). Additionally, the F/B ratio was similar (*P* > 0.05) in the duodenum and ileum between the two groups. However, compared with the GRY group, the F/B ratio of the GNY group exhibited greater (*P* < 0.05) in the jejunum (Figure 3B).

**TABLE 3:** Comparison of the relative abundance (%) of the representative bacteria at the phylum level in the small intestine of growth-retarded and normal yaks.

Items	GNY	GRY	SEM	*P*-value
Duodenum				
Bacteroidetes	42.15	48.36	1.539	0.198
Firmicutes	36.58	34.04	1.245	0.607
Proteobacteria	5.77	4.40	0.523	0.451
Acidobacteria	2.21	1.43	0.263	0.432
Chloroflexi	1.35	1.06	0.104	0.368
Actinobacteria	0.93	0.99	0.075	0.867
Planctomycetes	1.00	0.56	0.090	0.055
Epsilonbacteraeota	0.75	0.71	0.103	0.937
Spirochetes	0.96	0.94	0.190	0.961
Fibrobacteres	0.58	1.10	0.321	0.620
Tenericutes	0.49	0.65	0.083	0.572
Jejunum				
Firmicutes	38.94	15.18	3.767	< 0.001
Bacteroidetes	24.89	37.67	2.344	0.001
Proteobacteria	9.49	17.81	1.324	< 0.001
Acidobacteria	4.03	10.27	0.953	< 0.001
Planctomycetes	1.89	3.10	0.190	< 0.001
Chloroflexi	1.89	1.97	0.087	0.691
Actinobacteria	1.20	2.39	0.203	< 0.001
Gemmatimonadetes	0.35	0.79	0.075	< 0.001
Spirochetes	0.52	0.21	0.095	0.107
Kiritimatiellaeota	0.87	0.19	0.132	0.004
Ileum				
Firmicutes	35.01	38.03	1.398	0.338
Bacteroidetes	34.39	27.86	1.101	0.001
Proteobacteria	12.28	14.13	0.530	0.098
Acidobacteria	6.46	6.87	0.287	0.504
Planctomycetes	1.94	2.41	0.123	0.068
Chloroflexi	0.84	1.20	0.070	0.009
Actinobacteria	1.05	0.72	0.053	0.002
Spirochetes	1.43	0.17	0.200	0.001
Gemmatimonadetes	0.35	0.55	0.044	0.032

*GNY, growth normal yak (n = 6); GRY, growth-retarded yak (n = 6); SEM, standard error of the mean. Phylum with the average relative abundance was ≥ 0.5% in at least one group.*

**FIGURE 5:**
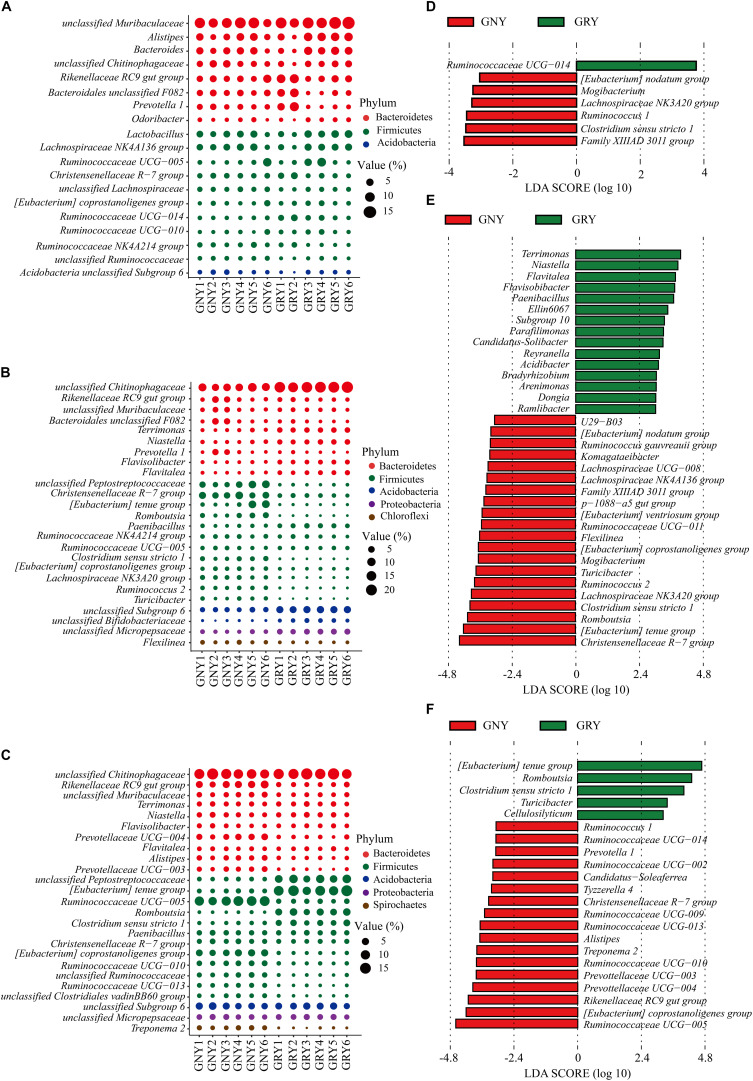
Taxa plots depicting bacterial composition in the small intestine of GNY (*n* = 6) and GRY (*n* = 6) groups. Bubble plot showing the relative abundance of dominant bacteria at genus level in the duodenum **(A)**, jejunum **(B)**, and ileum **(C)**. Genus with the average relative abundance was ≥ 1% in at least one group. Linear discriminant analysis effect size analysis identified the most differentially abundant genera in the duodenum **(D)**, jejunum **(E)**, and ileum **(F)** between GNY (red) and GRY (green) groups. Genus with linear discriminant analysis values higher than 3.5 is displayed. GNY, growth normal yak; GRY, growth-retarded yak.

At the genus level (Figures 5A–C and Supplementary Table 5), results showed that predominant genera in the small intestine of yaks included *Rikenellaceae RC9 gut* group, *Lactobacillus*, *Christensenellaceae R-7* group, and *Ruminococcaceae UCG-005*, as well as those unclassified bacteria derived from *Muribaculaceae*, *Chitinophagaceae*, and *Peptostreptococcaceae*. In the jejunum, compared with the GNY group, the bacteria, including *unclassified Chitinophagaceae*, *Terrimonas*, *Niastella*, *Flavisolibacter*, *Flavitalea*, *Paenibacillus*, *unclassified Micropepsaceae*, *unclassified Subgroup 6* (Acidobacteria), and *unclassified Bifidobacteriaceae*, were significantly enriched (*P* < 0.05) in the GRY group. However, the *unclassified Peptostreptococcaceae*, *Christensenellaceae R-7 group*, *Romboutsia*, *Clostridium sensu stricto 1*, *(Eubacterium) coprostanoligenes group*, *Lachnospiraceae NK3A20 group*, *Ruminococcus 2*, *Turicibacter*, and *Flexilinea* were significantly enriched (*P* < 0.05) in the GNY group. In the ileum, the relative abundances of *Rikenellaceae RC9 gut* group, *unclassified Muribaculaceae*, *Prevotellaceae UCG-004*, *Alistipes*, *Prevotellaceae UCG-003*, *Ruminococcaceae UCG-005*, *(Eubacterium) coprostanoligenes group*, *Ruminococcaceae UCG-010*, *unclassified Ruminococcaceae*, *Ruminococcaceae UCG-013*, and *unclassified Clostridiales vadinBB60* group were higher (*P* < 0.05) in the GNY group than those in the GRY group. However, the relative abundances of *unclassified Peptostreptococcaceae*, *(Eubacterium) tenue group*, *Romboutsia*, and *Clostridium sensu stricto 1* displayed an opposite trend. In the duodenum, jejunum, and ileum, 7, 35, and 22 differential bacteria were observed between the two groups, respectively. Of these, the *Ruminococcaceae UCG-014*, *Christensenellaceae R-7* group, and *Ruminococcaceae UCG-005* showed the highest difference in the corresponding gastrointestinal regions between the two groups (Figures 5D–F).

### Difference of Bacterial Community in the Large Intestine Between Growth-Retarded and Normal Yaks

In the large intestine, a total of 32 bacterial phyla were identified in 24 digesta samples. The number of bacteria phyla identified in the cecum and colon of GNY and GRY groups were 26 and 27 as well as 27 and 25, respectively. The majority of the bacteria belonged to the Firmicutes (cecum: GNY = 57.32% and GRY = 53.84%; colon: GNY = 54.72% and GRY = 54.09%), Bacteroidetes (cecum: GNY = 32.72% and GRY = 32.75%; colon: GNY = 32.69% and GRY = 28.92%), and Proteobacteria (cecum: GNY = 1.94% and GRY = 1.72%; colon: GNY = 2.15% and GRY = 1.52%), which accounted for appropriately 90% of all bacterial taxa. In the cecum, the relative abundances of Tenericutes (*P* = 0.090) and Actinobacteria (*P* = 0.068) were slightly higher (*P* < 0.05) in the GRY group as compared with the GNY group. In the colon, the relative abundance of Spirochetes in the GNY group was slightly higher (*P* = 0.092) than that in the GRY group (Table 4). Moreover, no significant difference (*P* > 0.05) in the F/B ratio was observed between the two groups (Figure 3C).

**TABLE 4:** Comparison of the relative abundance (%) of the representative bacteria at the phylum level in the large intestine of growth-retarded and normal yaks.

Items	GNY	GRY	SEM	*P*-value
Cecum				
Firmicutes	57.32	53.84	1.825	0.510
Bacteroidetes	32.72	32.75	1.688	0.992
Proteobacteria	1.94	1.27	0.194	0.191
Spirochetes	1.11	1.28	0.203	0.818
Tenericutes	0.78	1.10	0.079	0.090
Actinobacteria	0.53	0.86	0.078	0.068
Chloroflexi	0.46	0.97	0.193	0.355
Colon				
Firmicutes	54.72	54.09	1.838	0.874
Bacteroidetes	32.69	28.92	2.973	0.631
Proteobacteria	2.15	1.52	0.228	0.728
Spirochetes	1.51	0.40	0.259	0.092
Tenericutes	1.16	1.65	0.273	0.630
Actinobacteria	0.53	0.89	0.134	0.501
Chloroflexi	0.41	1.03	0.315	0.694
Kiritimatiellaeota	0.19	0.55	0.199	0.532

*GNY, growth normal yak (n = 6); GRY, growth-retarded yak (n = 6); SEM, standard error of the mean. Phylum with the average relative abundance was ≥ 0.5% in at least one group.*

**FIGURE 6:**
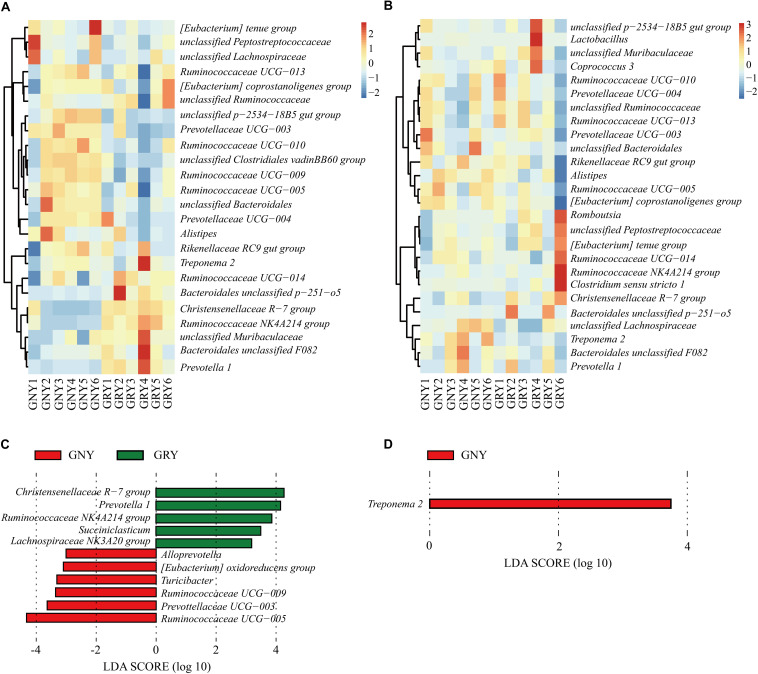
Taxa plots depicting bacterial composition in the large intestine of GNY (*n* = 6) and GRY (*n* = 6) groups. Heat map showing the relative abundance of dominant bacteria at genus level in the cecum **(A)** and colon **(B)**. Genus with the average relative abundance was ≥ 1% in at least one group. Linear discriminant analysis effect size analysis identified the most differentially abundant genera in the cecum **(C)** and colon **(D)** between GNY (red) and GRY (green) groups. Genus with linear discriminant analysis values higher than 3.5 is displayed. GNY, growth normal yak; GRY, growth-retarded yak.

At the genus level (Figures 6A,B and Supplementary Table 6), *Ruminococcaceae UCG-005* was the most dominant bacterium in the large intestine of the GNY (cecum, 14.94%; colon, 12.72%) and GRY (cecum, 10.38%; colon, 7.89%) groups, followed by *Rikenellaceae RC9 gut* group (cecum: GNY = 7.94% and GRY = 7.67%; colon: GNY = 8.18% and GRY = 6.26%), *(Eubacterium) coprostanoligenes* group (cecum: GNY = 5.47% and GRY = 5.57%; colon: GNY = 4.53% and GRY = 3.66%), *Ruminococcaceae UCG-010* (cecum: GNY = 6.90% and GRY = 4.25%; colon: GNY = 4.11% and GRY = 3.70%), and *Christensenellaceae R-7* group (cecum: GNY = 2.54% and GRY = 6.14%; colon: GNY = 4.12% and GRY = 5.33%). In the cecum, the relative abundances of *Bacteroidales unclassified p-2534-18B5 gut* group, *Prevotellaceae UCG-003*, *unclassified Clostridiales vadinBB60* group, and *Ruminococcaceae UCG-009* in the GNY group were higher (*P* < 0.05) than those in the GRY group. Furthermore, the relative abundances of *unclassified Bacteroidales* (*P* = 0.064), *Ruminococcaceae UCG-005* (*P* = 0.062), and *Ruminococcaceae UCG-010* (*P* = 0.072) tended to be higher in the GNY group than the GRY group. In the colon, compared with the GRY group, the *unclassified Lachnospiraceae* (*P* = 0.063) and *Treponema 2* (*P* = 0.052) were slightly higher in the GNY group. The LEfSe analysis revealed that 11 and 1 differential bacteria were identified in the cecum and colon, respectively, between the two groups. In the cecum, the *Ruminococcaceae UCG-005* and *Christensenellaceae R-7 group* showed a higher difference between the two groups. The differential bacteria were *Treponema 2* in the colon (Figures 6C,D).

## Discussion

Although they have no major clinical problems, the yaks with retardation are related to higher morbidity and mortality and lower feed efficiency, which result in increased feeding costs. The healthy development of GIT plays an essential role in the growth process of animals (Malmuthuge et al., 2015; Xiao et al., 2016). In humans, studies have demonstrated that the microbial composition in the GIT of stunted children was different from that of healthy children of the same age (Subramanian et al., 2014; Blanton et al., 2016). In the present study, we compared the bacterial community in the GIT (rumen, duodenum, jejunum, ileum, cecum, and colon) of GRYs and GNYs. The current study showed that the phyla Bacteroidetes and Firmicutes were the dominant bacteria of GIT in GRYs and GNYs. Consistent with our study, the two phyla were also found to be presented abundantly in the GIT of dairy cattle (Mao et al., 2015), goats (Li et al., 2019), yaks (Cui et al., 2020b), and steers (de Oliveira et al., 2013), suggesting the functional importance of Bacteroidetes and Firmicutes in the GIT of ruminants. In addition, the PCoA results revealed that the bacterial composition and structure in each segment of GIT were distinct between the GNY and GRY groups. Similar to our results, Hu et al. (2019) and Che et al. (2019) have reported that the composition of bacteria in the GIT between growth-retarded and normal animals was different.

In ruminants, the rumen, which harbors a great deal of inhabiting microbes, is typically the main digestive and absorptive organ for VFA and excess ammonia-N (Henderson et al., 2015). A previous study reported that Firmicutes had an important role in the process of energy absorption (Ley et al., 2006). Additionally, Firmicutes are known to be involved in the degradation of oligosaccharides, starch, and cellulose (Ahmad et al., 2020). In the rumen of yaks, the research found that the relative abundance of Firmicutes in the feeding group was significantly increased as compared with the grazing group (Zhou et al., 2017). Zou et al. (2019) reported that grazing yaks with low feed efficiency exhibited lower Firmicutes relative abundance in the rumen. Consistent with previous studies, in the current study, the relative abundance of Firmicutes in the GNY group was slightly higher than that in the GRY group, suggesting that GNYs had better feed efficiency. Moreover, Jia et al. (2018) found that the increased Tenericutes relative abundance in the rumen of fattening lambs was related to higher nitrogen utilization. Our study revealed that the relative abundance of Tenericutes in the GNY group was slightly higher than that in the GRY group, indicating that the nitrogen utilization of GNYs was higher. In the future, more experiments should be conducted to investigate the potential effects of those bacteria on the growth performance of yaks.

Our study revealed that the genera *Rikenellaceae RC9 gut* group, *Prevotella*, and *Christensenellaceae R-7* group were the predominant bacteria in the rumen, which were in accordance with previous findings in yaks (Xue et al., 2018; Hu et al., 2019). The bacteria belonging to the *Christensenellaceae* family can secrete α-arabinosidase, β-glucosidase, and β-galactosidase, which are related to feed efficiency (Perea et al., 2017). A previous study reported that the relative abundance of *Christensenellaceae* was inversely associated with BW (Waters and Ley, 2019). In the present study, the *Christensenellaceae R-7* group relative abundance in the GRY group was higher than that in the GNY group. The decreased relative abundance of *Christensenellaceae* may increase the BW of yaks. In the rumen, the bacteria from *Ruminococcaceae* family play a vital role in fiber degradation and biohydrogenation (Gagen et al., 2015; Opdahl et al., 2018). A study has found that the relative abundance of *Ruminococcaceae* was higher in the rumen of beef cattle with higher feed efficiency (Li and Guan, 2017). Results from the current study showed that the relative abundances of *Ruminococcaceae NK4A214* group and *Ruminococcaceae UCG-014* were significantly higher in the GNY group than those in the GRY group, indicating that GNYs had a higher ability of degradation for fiber. In addition, compared with the GRY group, the relative abundance of *Treponema 2* was higher in the GNY group. *Treponema* is mainly involved in the utilization of soluble carbohydrates (Stanton and Canale-Parola, 1980). Besides, *Treponema* showed a higher abundance in the rumen of beef cattle when the high concentrate diets was fed (Chen et al., 2011). The results from ruminal bacteria indicated that the fiber utilization of GRYs was lower as compared with GNYs.

The microbiota in the gut plays an important role in the host by absorbing essential nutrients, promoting the development of the GIT, and maintaining immune function (Guzman et al., 2015; Yeoman et al., 2018). The balanced intestinal microbiota is essential for animals to grow normally (Alipour et al., 2018). In the jejunum, we found that the F/B ratio was strikingly higher in the GNY group than that in the GRY group. In previous research, the higher F/B ratio in the gut was confirmed to be associated with human obesity (Turnbaugh et al., 2006). A study in cattle found that the increased F/B ratio was positively correlated with growth rate and feed efficiency (Myer et al., 2015a), suggesting the important role of Firmicutes and Bacteroidetes in energy metabolism. The results from our study indicated that GNYs have a higher ability to utilize the crude fiber of roughage, thereby providing more energy for the host. Moreover, a study in humans reported that the abundances of Proteobacteria and Acidobacteria were markedly increased in cases that suffered from enteritis (Wang et al., 2018). In the current study, the phyla Proteobacteria and Acidobacteria in the jejunum of the GRY group were obviously higher as compared with the GNY group. Besides, according to our data (not yet published), the GRYs showed higher mRNA expression of inflammatory cytokines in the jejunum and concentrations of inflammatory cytokines in the serum as compared with GNYs. These results might indicate that the GRYs had intestinal inflammation. However, these results need future investigation. In the jejunum and ileum, the relative abundance of Planctomycetes in the GRY group was higher than that in the GNY group. A previous study in marine microorganisms found that the Planctomycetes could utilize nitrite and ammonium ions to generate nitrogen gas for energy under anoxic conditions (Delmont et al., 2018). However, the potential function of Planctomycetes in animals still needs further elucidation.

At the genus level, we found that the *unclassified Muribaculaceae* and *unclassified Chitinophagaceae* were the predominant bacteria in the duodenum, jejunum, and ileum. Compared with the duodenum, the jejunum and ileum showed a larger difference of bacterial community between the GNY and GRY groups. In the jejunum, the relative abundance of *unclassified Chitinophagaceae* in the GRY group was higher than that in the GNY group. A previous study has reported that *Chitinophagaceae* harbored several enzymes that could degrade fungal cell wall, including endoglucanases, b-glucanases, and chitinases (Carrión et al., 2019). The fungi have an important role in maintaining health for the host (Bernardes et al., 2020). The results from our study suggested that higher *Chitinophagaceae* abundance in the gut might have negative effects on GRYs. Interestingly, in the current study, the proportion of *unclassified Peptostreptococcaceae* and *(Eubacterium) tenue* group exhibited an opposite trend in the jejunum and ileum between the two groups. Previous research showed that the genus *unclassified Peptostreptococcaceae* and *Eubacterium* might have a vital role in feed digestion (Mao et al., 2012). The results implied that intestinal regions showed strong determinants of microbial community structure and function. In a human study, the *Rikenellaceae* can be used as a microbial marker to the microflora of obese adolescents (Del Chierico et al., 2018). Our study showed that the relative abundance of *Rikenellaceae RC9 gut* group in the ileum of the GNY group was higher than that of the GRY group. A recent finding reported that compared with polyp-associated intestinal tissue, the *Romboutsia* genus was more abundant in the healthy intestine (Mangifesta et al., 2018), which indicated the important role of *Romboutsia* in maintaining the health of the gut. However, the relative abundance of *Romboutsia* in the jejunum and ileum exhibited distinct results between the GNY and GRY groups. The possible reason is that the jejunum and ileum have different functions. The families *Clostridiaceae* and *Prevotellaceae* in the small intestine have been confirmed to be important in carbohydrate metabolism, and those bacteria might increase the ability of the intestine for nutrient acquisition, as has been reported in humans (Zoetendal et al., 2012). In the ileum, the *Prevotellaceae UCG-004* and *unclassified Clostridiales vadinBB60* group proportions in the GNY group were higher than those in the GRY group, indicating that the GNYs had higher efficiency of carbohydrate metabolism. Moreover, we also found that the genera *Terrimonas*, *Niastella*, *Flavisolibacter*, *Flavitalea*, *Paenibacillus*, *Turicibacter*, and *Flexilinea* displayed significant differences in the gut between the GNY and GRY groups. Unfortunately, at present, the knowledge of microflora is limited. However, there is no denying that the bacterial community in the small intestine between GRYs and GNYs is distinct.

In addition to the rumen and small intestine, the large intestine also has an important function in the normal growth of yaks. The cecum and colon are important parts of immune systems; besides, the large intestine plays an essential role in the post-ruminal degradation of cellulose and starch, which are considered to be vital in digestion (Armstrong and Smithard, 1979). A previous study reported that the relative abundances of *Ruminococcaceae*, *Lachnospiraceae*, and *Clostridiaceae* in the cecum of steers with high feed efficiency were higher (Myer et al., 2015b). The members of *Ruminococcaceae* are known for higher cellulolytic capacity. Consistent with rumen, the GNY group had higher *Ruminococcaceae* relative abundance. Moreover, the relative abundances of *Prevotellaceae UCG-003*, *Treponema 2*, and *unclassified Lachnospiraceae* in the large intestine of the GNY group were higher than those of the GRY group. These microorganisms may also contribute to further downstream feed fermentation. Overall, the microbial difference of the large intestine between the GNY and GRY groups was relatively low as compared with the rumen and small intestine.

## Conclusion

In the current study, the GIT showed different bacterial communities between GRYs and GNYs. The relative abundances of bacteria related to oligosaccharide, starch, and cellulose degradation, including *Ruminococcaceae*, *Treponema*, *Clostridiaceae*, *Prevotellaceae*, and *Lachnospiraceae*, in the GIT of GNYs were higher than those of GRYs. Moreover, compared with GNYs, the relative abundances of some bacteria, including *Christensenellaceae R-7* group, *unclassified Chitinophagaceae*, and *(Eubacterium) tenue* group, in the GIT of GRYs were higher. Results in our study revealed that the bacterial community in the GIT of GRYs was disrupted. Regulating the microbial community may be an effective solution to promote the compensatory growth of GRYs.

## Data Availability Statement

The sequences from the current study have been deposited in the Sequence Read Archive database of National Center for Biotechnology Information with the accession number PRJNA659573.

## Ethics Statement

This animal experiment was carried out in accordance with the Regulation on the Administration of Laboratory Animals (2017 Revision) promulgated by Decree No. 676 of the State Council. The protocol was authorized and approved by the Institutional Animal Care and Use Committee of Sichuan Agricultural University (Chengdu, Sichuan, China).

## Author Contributions

JM, ZW, and SZ conceived and designed the research. JM, YZ, XW, GC, HZ, QP, BX, LW, and XK performed the animal experiment and sample collection. JM, YZ, and RH analyzed the data. JM wrote the original manuscript. JM, YZ, ZW, XY, and AS reviewed the manuscript. All authors read and approved the final manuscript.

## Conflict of Interest

The authors declare that the research was conducted in the absence of any commercial or financial relationships that could be construed as a potential conflict of interest.

## Footnotes

1 http://www.ehbio.com/ImageGP

2 http://huttenhower.sph.harvard.edu/galaxy
