# Supplementary material for: Comparing the Bacterial Community in the Gastrointestinal Tracts Between Growth-Retarded and Normal Yaks on the Qinghai–Tibetan Plateau
Source: Front Microbiol. 2020 Dec 18;11:600516. doi: 10.3389/fmicb.2020.600516 (PMC7775487; doi:10.3389/fmicb.2020.600516)
Supplement: Supplementary file 1 [file Data_Sheet_1.docx]

Supplementary Material

# SUPPLEMENTARY TABLES

**TABLE 1 |** The detailed body weight of yaks used in the current study.

| **Groups** | **Animal no.** | **Lot no.** | **Body weight (kg)** |
| --- | --- | --- | --- |
| GNY | 0602 | GNY-1 | 110 |
|  | 0689 | GNY-2 | 107 |
|  | 0680 | GNY-3 | 112 |
|  | 0691 | GNY-4 | 114 |
|  | 0681 | GNY-5 | 110 |
|  | 0682 | GNY-6 | 114 |
|  | 0690 | GNY-7 | 119 |
|  | 0679 | GNY-8 | 107 |
| GRY | 0711 | GRY-1 | 74 |
|  | 0767 | GRY-2 | 81 |
|  | 0797 | GRY-3 | 67 |
|  | 0708 | GRY-4 | 72 |
|  | 0793 | GRY-5 | 73 |
|  | 0731 | GRY-6 | 74 |
|  | 0799 | GRY-7 | 85 |
|  | 0784 | GRY-8 | 66 |

GNY, growth normal yak (n = 8); GRY, growth-retarded yak (n = 8). The serial number of yaks from 1 to 6 were used to slaughter.

**TABLE 2 |** Data acquisition of all samples.

| **Items** | **Samples no.** | **Raw sequences** | **Effective sequences** | **Sequencing length** | **OTUs** | **Q30%** |
| --- | --- | --- | --- | --- | --- | --- |
| Rumen | GNY-1 | 34344 | 32909 | 296 | 2452 | 93.1% |
|  | GNY-2 | 33343 | 31855 | 296 | 2404 | 93.9% |
|  | GNY-3 | 30185 | 28647 | 297 | 2039 | 93.7% |
|  | GNY-4 | 30931 | 29347 | 296 | 2302 | 94.8% |
|  | GNY-5 | 33806 | 32294 | 293 | 2020 | 94.1% |
|  | GNY-6 | 33184 | 31616 | 295 | 1965 | 94.1% |
|  | GRY-1 | 31438 | 30070 | 298 | 2383 | 93.3% |
|  | GRY-2 | 39364 | 37833 | 297 | 2056 | 93.8% |
|  | GRY-3 | 35130 | 33615 | 296 | 2408 | 94.6% |
|  | GRY-4 | 30044 | 28970 | 295 | 1999 | 93.8% |
|  | GRY-5 | 35670 | 34088 | 297 | 1845 | 94.6% |
|  | GRY-6 | 37575 | 35847 | 301 | 1733 | 93.7% |
| Duodenum | GNY-1 | 37715 | 36014 | 298 | 2927 | 93.0% |
|  | GNY-2 | 34958 | 33331 | 295 | 2815 | 94.9% |
|  | GNY-3 | 36287 | 34825 | 296 | 2949 | 93.7% |
|  | GNY-4 | 32472 | 30672 | 297 | 2677 | 94.0% |
|  | GNY-5 | 36516 | 34711 | 297 | 2145 | 94.3% |
|  | GNY-6 | 31397 | 29780 | 295 | 2532 | 94.5% |
|  | GRY-1 | 31853 | 30217 | 293 | 2570 | 93.3% |
|  | GRY-2 | 36321 | 34934 | 292 | 2020 | 93.8% |
|  | GRY-3 | 30523 | 29141 | 298 | 2164 | 93.6% |
|  | GRY-4 | 36367 | 34804 | 298 | 2383 | 94.1% |
|  | GRY-5 | 39303 | 37393 | 303 | 2673 | 94.4% |
|  | GRY-6 | 39919 | 38002 | 302 | 2543 | 93.1% |
| Jejunum | GNY-1 | 33599 | 32173 | 298 | 2446 | 94.6% |
|  | GNY-2 | 34111 | 32635 | 296 | 2353 | 93.1% |
|  | GNY-3 | 36071 | 34388 | 297 | 2632 | 94.2% |
|  | GNY-4 | 39091 | 37434 | 298 | 2278 | 94.4% |
|  | GNY-5 | 35702 | 33643 | 305 | 2432 | 93.6% |
|  | GNY-6 | 39492 | 37582 | 305 | 2270 | 94.0% |
|  | GRY-1 | 33419 | 31676 | 298 | 3445 | 93.6% |
|  | GRY-2 | 39165 | 37295 | 297 | 2946 | 94.9% |
|  | GRY-3 | 33283 | 31847 | 296 | 2672 | 93.3% |
|  | GRY-4 | 33836 | 32138 | 296 | 2607 | 93.2% |
|  | GRY-5 | 30537 | 28950 | 296 | 3099 | 93.9% |
|  | GRY-6 | 30330 | 29057 | 296 | 2823 | 94.6% |
| Ileum | GNY-1 | 38847 | 37394 | 310 | 2155 | 93.3% |
|  | GNY-2 | 34928 | 33350 | 317 | 2435 | 94.3% |
|  | GNY-3 | 32041 | 30432 | 311 | 2168 | 93.9% |
|  | GNY-4 | 37394 | 35500 | 308 | 2562 | 93.5% |
|  | GNY-5 | 36951 | 35082 | 306 | 2202 | 94.7% |
|  | GNY-6 | 33482 | 32028 | 304 | 2161 | 93.6% |
|  | GRY-1 | 36472 | 34954 | 297 | 2574 | 93.8% |
|  | GRY-2 | 31324 | 29582 | 297 | 2250 | 94.5% |
|  | GRY-3 | 32243 | 30960 | 297 | 2191 | 94.3% |
|  | GRY-4 | 37758 | 35816 | 297 | 2553 | 94.5% |
|  | GRY-5 | 39112 | 37208 | 297 | 2390 | 94.2% |
|  | GRY-6 | 32051 | 30618 | 297 | 2256 | 93.3% |
| Cecum | GNY-1 | 30203 | 28656 | 297 | 2058 | 93.0% |
|  | GNY-2 | 30762 | 29134 | 295 | 1902 | 94.6% |
|  | GNY-3 | 30850 | 29331 | 296 | 1977 | 94.5% |
|  | GNY-4 | 30038 | 28810 | 296 | 2005 | 93.7% |
|  | GNY-5 | 32118 | 30401 | 295 | 1904 | 94.2% |
|  | GNY-6 | 34150 | 32702 | 296 | 2035 | 93.8% |
|  | GRY-1 | 31565 | 30338 | 295 | 2324 | 93.9% |
|  | GRY-2 | 34914 | 33170 | 296 | 2281 | 93.4% |
|  | GRY-3 | 30703 | 29496 | 296 | 2548 | 93.2% |
|  | GRY-4 | 35708 | 33875 | 297 | 2292 | 93.1% |
|  | GRY-5 | 30522 | 29322 | 295 | 2629 | 94.7% |
|  | GRY-6 | 37910 | 36110 | 296 | 2439 | 94.2% |
| Colon | GNY-1 | 33624 | 31969 | 297 | 1657 | 94.8% |
|  | GNY-2 | 36434 | 34872 | 298 | 1991 | 93.1% |
|  | GNY-3 | 35949 | 33946 | 297 | 2304 | 94.7% |
|  | GNY-4 | 35564 | 33732 | 298 | 2316 | 93.0% |
|  | GNY-5 | 39976 | 38162 | 298 | 1816 | 94.1% |
|  | GNY-6 | 31883 | 30137 | 297 | 2239 | 94.9% |
|  | GRY-1 | 33491 | 31789 | 294 | 1627 | 94.1% |
|  | GRY-2 | 31498 | 30266 | 299 | 2370 | 94.6% |
|  | GRY-3 | 34354 | 32861 | 298 | 1689 | 94.1% |
|  | GRY-4 | 32175 | 30754 | 297 | 1257 | 93.4% |
|  | GRY-5 | 36026 | 34356 | 298 | 2292 | 94.7% |
|  | GRY-6 | 36469 | 34608 | 296 | 1265 | 93.5% |

GNY, growth normal yak (n = 6); GRY, growth-retarded yak (n = 6); OTUs, operational taxonomic units.

**TABLE 3 |** Analysis of permutational multivariate analysis of variance results of bacterial community according to gastrointestinal regions between growth-retarded and normal yaks.

| **Regions** | **Groups** | **R^2^** | ***P*-value** |
| --- | --- | --- | --- |
| Rumen | GNY vs. GRY | 0.218 | 0.003 |
| Duodenum | GNY vs. GRY | 0.183 | 0.010 |
| Jejunum | GNY vs. GRY | 0.557 | 0.003 |
| Ileum | GNY vs. GRY | 0.594 | 0.003 |
| Cecum | GNY vs. GRY | 0.304 | 0.003 |
| Colon | GNY vs. GRY | 0.144 | 0.051 |

GNY, growth normal yak (n = 6); GRY, growth-retarded yak (n = 6). *P* < 0.05 means a significant difference of the bacterial structure in each corresponding regions of the gastrointestinal tracts between GNY and GRY groups.

**TABLE 4 |** Comparison of the relative abundance (%) of the representative bacteria at the genus level in the rumen of growth-retarded and normal yaks.

| **Items** | | **GNY** | **GRY** | **SEM** | ***P*-value** |
| --- | --- | --- | --- | --- | --- |
| Bacteroidetes | *Rikenellaceae RC9 gut group* | 13.94 | 13.95 | 0.924 | 0.993 |
|  | *Bacteroidales unclassified F082* | 8.56 | 10.23 | 0.685 | 0.411 |
|  | *Prevotella 1* | 6.22 | 6.19 | 0.409 | 0.993 |
|  | *unclassified Muribaculaceae* | 2.97 | 4.02 | 0.639 | 0.557 |
|  | *Bacteroidales unclassified p-251-o5* | 3.90 | 2.14 | 0.817 | 0.444 |
|  | *Prevotellaceae UCG-003* | 1.67 | 1.69 | 0.175 | 0.993 |
|  | *unclassified Bacteroidales UCG-001* | 1.78 | 0.76 | 0.238 | 0.084 |
| Firmicutes | *Christensenellaceae R-7 group* | 6.17 | 9.40 | 0.556 | 0.001 |
|  | *Ruminococcaceae NK4A214 group* | 4.34 | 2.76 | 0.376 | 0.046 |
|  | *Succiniclasticum* | 3.35 | 2.80 | 0.248 | 0.456 |
|  | *[Eubacterium] coprostanoligenes group* | 2.15 | 1.71 | 0.130 | 0.152 |
|  | *Lachnospiraceae NK3A20 group* | 1.34 | 1.16 | 0.141 | 0.661 |
|  | *Ruminococcaceae UCG-014* | 1.78 | 1.05 | 0.147 | 0.038 |
|  | *Ruminococcaceae UCG-005* | 1.85 | 1.32 | 0.182 | 0.323 |
|  | *Ruminococcaceae UCG-010* | 1.75 | 1.34 | 0.130 | 0.187 |
|  | *unclassified Lachnospiraceae* | 1.20 | 1.02 | 0.093 | 0.502 |
|  | *Ruminococcaceae UCG-011* | 1.18 | 1.01 | 0.061 | 0.213 |
| Spirochaetes | *Treponema 2* | 3.36 | 1.73 | 0.352 | 0.026 |
| Chloroflexi | *Flexilinea* | 0.95 | 1.66 | 0.203 | 0.147 |

GNY, growth normal yak (n = 6); GRY, growth-retarded yak (n = 6); SEM, standard error of the mean. The genus with the average relative abundance was ≥ 1% in at least one group.

**TABLE 5 |** Comparison of the relative abundance (%) of the representative bacteria at the genus level in the small intestine of growth-retarded and normal yaks.

| **Items** | | **GNY** | **GRY** | **SEM** | ***P*-value** |
| --- | --- | --- | --- | --- | --- |
| Duodenum | | | | | |
| Bacteroidetes | *unclassified* *Muribaculaceae* | 11.30 | 14.65 | 1.012 | 0.370 |
|  | *Alistipes* | 4.34 | 5.84 | 0.624 | 0.571 |
|  | *Bacteroides* | 3.96 | 4.64 | 0.540 | 0.782 |
|  | *unclassified Chitinophagaceae* | 3.55 | 2.96 | 0.349 | 0.704 |
|  | *Rikenellaceae RC9 gut group* | 3.87 | 5.31 | 0.620 | 0.531 |
|  | *Bacteroidales unclassified F082* | 3.18 | 3.40 | 0.575 | 0.937 |
|  | *Prevotella 1* | 2.33 | 3.06 | 0.674 | 0.895 |
|  | *Odoribacter* | 0.94 | 1.01 | 0.126 | 0.937 |
| Firmicutes | *Lactobacillus* | 3.35 | 4.08 | 0.403 | 0.727 |
|  | *Lachnospiraceae NK4A136 group* | 3.54 | 3.47 | 0.339 | 0.922 |
|  | *Ruminococcaceae UCG-005* | 2.18 | 2.91 | 0.776 | 0.937 |
|  | *Christensenellaceae R-7 group* | 2.09 | 2.25 | 0.215 | 0.893 |
|  | *unclassified* *Lachnospiraceae* | 1.76 | 1.28 | 0.104 | 0.114 |
|  | *[Eubacterium] coprostanoligenes group* | 1.36 | 1.50 | 0.201 | 0.846 |
|  | *Ruminococcaceae UCG-014* | 0.98 | 2.15 | 0.267 | 0.126 |
|  | *Ruminococcaceae UCG-010* | 1.01 | 1.29 | 0.174 | 0.698 |
|  | *Ruminococcaceae NK4A214 group* | 1.62 | 1.03 | 0.180 | 0.280 |
|  | *unclassified Ruminococcaceae* | 1.21 | 0.68 | 0.100 | 0.038 |
| Acidobacteria | *Acidobacteria unclassified Subgroup 6* | 1.24 | 0.81 | 0.144 | 0.337 |
| Jejunum | | | | | |
| Bacteroidetes | *unclassified* *Chitinophagaceae* | 8.34 | 18.97 | 1.678 | <0.001 |
|  | *Rikenellaceae RC9 gut group* | 2.46 | 1.52 | 0.388 | 0.270 |
|  | *unclassified Muribaculaceae* | 1.90 | 1.38 | 0.226 | 0.283 |
|  | *Bacteroidales unclassified F082* | 2.21 | 0.83 | 0.441 | 0.144 |
|  | *Terrimonas* | 1.01 | 2.70 | 0.266 | <0.001 |
|  | *Niastella* | 1.12 | 2.48 | 0.221 | <0.001 |
|  | *Prevotella 1* | 1.71 | 0.52 | 0.382 | 0.141 |
|  | *Flavisolibacter* | 0.75 | 1.77 | 0.160 | <0.001 |
|  | *Flavitalea* | 0.68 | 1.77 | 0.181 | <0.001 |
| Firmicutes | *unclassified* *Peptostreptococcaceae* | 4.96 | 0.51 | 0.809 | 0.001 |
|  | *Christensenellaceae R-7 group* | 5.40 | 1.48 | 0.654 | <0.001 |
|  | *[Eubacterium] tenue group* | 3.34 | 0.24 | 0.833 | 0.072 |
|  | *Romboutsia* | 2.17 | 0.11 | 0.378 | 0.001 |
|  | *Paenibacillus* | 1.00 | 2.01 | 0.175 | <0.001 |
|  | *Ruminococcaceae NK4A214 group* | 1.55 | 1.13 | 0.109 | 0.064 |
|  | *Ruminococcaceae UCG-005* | 1.26 | 1.18 | 0.051 | 0.438 |
|  | *Clostridium sensu stricto 1* | 1.73 | 0.10 | 0.268 | <0.001 |
|  | *[Eubacterium] coprostanoligenes group* | 1.38 | 0.63 | 0.121 | <0.001 |
|  | *Lachnospiraceae NK3A20 group* | 1.61 | 0.22 | 0.222 | <0.001 |
|  | *Ruminococcus 2* | 1.26 | 0.18 | 0.167 | <0.001 |
|  | *Turicibacter* | 1.03 | 0.07 | 0.164 | <0.001 |
| Acidobacteria | *unclassified Subgroup 6* | 2.42 | 6.34 | 0.599 | <0.001 |
| Proteobacteria | *unclassified* *Micropepsaceae* | 1.38 | 3.24 | 0.298 | <0.001 |
| Chloroflexi | *Flexilinea* | 1.27 | 0.62 | 0.131 | 0.007 |
| Actinobacteria | *unclassified* *Bifidobacteriaceae* | 0.16 | 1.31 | 0.194 | <0.001 |
| Ileum | | | | | |
| Bacteroidetes | *unclassified Chitinophagaceae* | 10.65 | 12.35 | 0.600 | 0.172 |
|  | *Rikenellaceae RC9 gut group* | 4.22 | 1.59 | 0.445 | <0.001 |
|  | *unclassified Muribaculaceae* | 1.94 | 1.30 | 0.125 | 0.003 |
|  | *Terrimonas* | 1.54 | 1.63 | 0.084 | 0.647 |
|  | *Niastella* | 1.69 | 1.75 | 0.092 | 0.758 |
|  | *Flavisolibacter* | 1.23 | 1.22 | 0.065 | 0.922 |
|  | *Prevotellaceae UCG-004* | 2.72 | 0.96 | 0.306 | <0.001 |
|  | *Flavitalea* | 1.16 | 1.11 | 0.061 | 0.761 |
|  | *Alistipes* | 1.81 | 0.86 | 0.163 | <0.001 |
|  | *Prevotellaceae UCG-003* | 1.66 | 0.38 | 0.215 | <0.001 |
| Firmicutes | *unclassified* *Peptostreptococcaceae* | 1.13 | 5.93 | 0.766 | <0.001 |
|  | *[Eubacterium] tenue group* | 1.35 | 10.39 | 1.449 | <0.001 |
|  | *Ruminococcaceae UCG-005* | 9.30 | 1.64 | 1.190 | <0.001 |
|  | *Romboutsia* | 0.40 | 4.17 | 0.591 | <0.001 |
|  | *Clostridium sensu stricto 1* | 0.81 | 2.72 | 0.322 | <0.001 |
|  | *Paenibacillus* | 1.83 | 1.87 | 0.072 | 0.822 |
|  | *Christensenellaceae R-7 group* | 1.71 | 1.28 | 0.103 | 0.029 |
|  | *[Eubacterium] coprostanoligenes group* | 3.82 | 0.68 | 0.485 | <0.001 |
|  | *Ruminococcaceae UCG-010* | 2.04 | 0.75 | 0.203 | <0.001 |
|  | *unclassified* *Ruminococcaceae* | 1.65 | 0.48 | 0.191 | <0.001 |
|  | *Ruminococcaceae UCG-013* | 1.26 | 0.34 | 0.144 | <0.001 |
|  | *unclassified* *Clostridiales vadinBB60 group* | 1.10 | 0.33 | 0.127 | <0.001 |
| Acidobacteria | *unclassified Subgroup 6* | 3.95 | 3.85 | 0.196 | 0.840 |
| Proteobacteria | *unclassified Micropepsaceae* | 2.11 | 2.26 | 0.100 | 0.493 |
| Spirochaetes | *Treponema 2* | 1.40 | 0.14 | 0.200 | <0.001 |

GNY, growth normal yak (n = 6); GRY, growth-retarded yak (n = 6); SEM, standard error of the mean. The genus with the average relative abundance was ≥ 1% in at least one group.

**TABLE 6 |** Comparison of the relative abundance (%) of the representative bacteria at the genus level in the large intestine of growth-retarded and normal yaks.

| **Items** | | **GNY** | **GRY** | **SEM** | ***P*-value** |
| --- | --- | --- | --- | --- | --- |
| Cecum | |  |  |  |  |
| Bacteroidetes | *Rikenellaceae RC9 gut group* | 7.94 | 7.67 | 0.682 | 0.892 |
|  | *Prevotellaceae UCG-004* | 4.53 | 3.04 | 0.619 | 0.328 |
|  | *unclassified* *Muribaculaceae* | 2.49 | 2.82 | 0.087 | 0.096 |
|  | *Alistipes* | 2.71 | 2.25 | 0.172 | 0.277 |
|  | *Bacteroidales unclassified F082* | 2.19 | 3.56 | 0.443 | 0.216 |
|  | *Bacteroidales unclassified p-2534-18B5 gut group* | 3.62 | 1.37 | 0.482 | 0.038 |
|  | *Prevotellaceae UCG-003* | 2.56 | 1.72 | 0.164 | 0.024 |
|  | *Prevotella 1* | 0.46 | 3.30 | 0.557 | 0.018 |
|  | *unclassified Bacteroidales* | 1.75 | 1.00 | 0.172 | 0.064 |
|  | *Bacteroidales unclassified p-251-o5* | 0.13 | 1.16 | 0.235 | 0.063 |
| Firmicutes | *Ruminococcaceae UCG-005* | 14.94 | 10.38 | 1.082 | 0.062 |
|  | *[Eubacterium] coprostanoligenes group* | 5.47 | 5.57 | 0.448 | 0.916 |
|  | *Ruminococcaceae UCG-010* | 6.90 | 4.25 | 0.650 | 0.072 |
|  | *Christensenellaceae R-7 group* | 2.54 | 6.14 | 0.650 | 0.012 |
|  | *unclassified Ruminococcaceae* | 2.26 | 2.60 | 0.180 | 0.434 |
|  | *Ruminococcaceae UCG-013* | 2.22 | 1.85 | 0.174 | 0.387 |
|  | *unclassified* *Clostridiales vadinBB60 group* | 2.31 | 0.94 | 0.291 | 0.036 |
|  | *unclassified Peptostreptococcaceae* | 2.05 | 1.24 | 0.254 | 0.216 |
|  | *Ruminococcaceae UCG-014* | 1.51 | 1.79 | 0.099 | 0.235 |
|  | *Ruminococcaceae NK4A214 group* | 1.05 | 2.46 | 0.246 | 0.003 |
|  | *unclassified Lachnospiraceae* | 1.27 | 1.15 | 0.056 | 0.382 |
|  | *[Eubacterium] tenue group* | 1.09 | 0.68 | 0.138 | 0.237 |
|  | *Ruminococcaceae UCG-009* | 1.03 | 0.56 | 0.099 | 0.043 |
| Spirochaetes | *Treponema 2* | 1.03 | 1.24 | 0.202 | 0.673 |
| Colon | | | | | |
| Bacteroidetes | *Rikenellaceae RC9 gut group* | 8.18 | 6.26 | 0.867 | 0.757 |
|  | *unclassified Muribaculaceae* | 2.46 | 3.60 | 0.583 | 0.700 |
|  | *Prevotellaceae UCG-004* | 2.86 | 3.06 | 0.646 | 0.959 |
|  | *Alistipes* | 3.33 | 2.28 | 0.315 | 0.617 |
|  | *Bacteroidales unclassified F082* | 3.43 | 2.13 | 0.608 | 0.728 |
|  | *Prevotellaceae UCG-003* | 2.50 | 1.92 | 0.346 | 0.584 |
|  | *Bacteroidales unclassified p-2534-18B5 gut group* | 1.56 | 1.90 | 0.484 | 0.925 |
|  | *Prevotella 1* | 2.05 | 1.71 | 0.604 | 0.899 |
|  | *unclassified Bacteroidales* | 1.35 | 0.68 | 0.268 | 0.653 |
|  | *Bacteroidales unclassified p-251-o5* | 0.26 | 1.76 | 0.561 | 0.721 |
| Firmicutes | *Ruminococcaceae UCG-005* | 12.72 | 7.89 | 1.401 | 0.719 |
|  | *[Eubacterium] coprostanoligenes group* | 4.53 | 3.66 | 0.331 | 0.653 |
|  | *Christensenellaceae R-7 group* | 4.12 | 5.33 | 0.435 | 0.750 |
|  | *Ruminococcaceae UCG-010* | 4.11 | 3.70 | 0.602 | 0.891 |
|  | *unclassified Peptostreptococcaceae* | 2.58 | 3.71 | 0.543 | 0.698 |
|  | *[Eubacterium] tenue group* | 1.86 | 2.25 | 0.317 | 0.736 |
|  | *Ruminococcaceae UCG-014* | 2.36 | 2.37 | 0.404 | 0.983 |
|  | *unclassified Ruminococcaceae* | 1.93 | 1.97 | 0.204 | 0.949 |
|  | *Ruminococcaceae NK4A214 group* | 1.64 | 2.99 | 0.740 | 0.592 |
|  | *Ruminococcaceae UCG-013* | 1.40 | 1.91 | 0.265 | 0.634 |
|  | *Romboutsia* | 0.95 | 1.65 | 0.244 | 0.811 |
|  | *Clostridium sensu stricto 1* | 0.76 | 1.46 | 0.370 | 0.601 |
|  | *unclassified Lachnospiraceae* | 1.12 | 0.84 | 0.073 | 0.063 |
|  | *Coprococcus 3* | 0.78 | 1.25 | 0.264 | 0.575 |
|  | *Lactobacillus* | 0.18 | 1.06 | 0.462 | 0.670 |
| Spirochaetes | *Treponema 2* | 1.44 | 0.37 | 0.246 | 0.052 |

GNY, growth normal yak (n = 6); GRY, growth-retarded yak (n = 6); SEM, standard error of the mean. The genus with the average relative abundance was ≥ 1% in at least one group.
